# Pancreatic Pseudocysts Transpapillary and Transmural Drainage

**DOI:** 10.1155/2000/43853

**Published:** 2000-01

**Authors:** E. Della Libera, E. S. Siqueira, M. Morais, M. R. S. Rohr, C. Q. Brant, J. C. Ardengh, A. P. Ferrari

**Affiliations:** 1 Endoscopy Unit Division of Gastroenterology Universidade Federal de São Paulo São Paulo Brazil; 2 Rua Pedro de Toledo 980, cj 66 São Paulo-SP CEP 04039-002 Brazil

## Abstract

*Background:* Pancreatic pseudocyst endoscopic drainage has been described as a good treatment option, with morbidity and mortality rates that are lower than surgery. The aim of our study is to describe the efficacy of different forms of endoscopic drainage and estimate pseudocyst recurrence rate after short follow up period.

*Patients and Methods:* We studied 30 patients with pancreatic pseudocyst that presented some indication for treatment: persistent abdominal pain, infection or cholestasis. Clinical evaluation was performed with a pain scale, 0 meaning absence of pain and 4 meaning continuous pain. Pseudocysts were first evaluated by abdominal CT scan, and after endoscopic retrograde pancreatography the patients were treated by transpapillary or transmural (cystduodenostomy or cystgastrostomy) drainage. Pseudocyst resolution was documented by serial CT scans.

*Results:* 25/30 patients could be treated. Drainage was successful in 21 (70% in an ‘intention to treat’ basis). After a mean follow-up of 42±35.82 weeks, there was only 1 (4.2%) recurrence. A total of 6 complications occurred in 37 procedures (16.2%), and all but 2 were managed clinically and/or endoscopically: there was no mortality related to the procedure. Patients submitted to combined drainage needed more procedures than the other groups. There was no difference in the efficacy when we compared the three different drainage methods.

*Conclusions:* We concluded that pancreatic pseudocyst endoscopic drainage is possible in most patients, with high success rate and low morbidity.


*HPB Surgery*, 2000, Vol. 11, pp. 333–338
Reprints available directly from the publisher
Photocopying permitted by license only

© 2000 OPA (Overseas Publishers Association) N.V.
Published by license under
the Harwood Academic Publishers imprint,
part of The Gordon and Breach Publishing Group.
Printed in Malaysia.

### *Clinical Series*

### Pancreatic Pseudocysts Transpapillary and Transmural Drainage

E. DELLA LIBERA [a], E. S. SIQUEIRA [a], M. MORAIS [a], M. R. S. ROHR [a], C. Q. BRANT [a], J. C. ARDENGH [a] and A. P. FERRARI [b,*]

[a] *Fellows of Gastroenterology, Endoscopy Unit;* [b] *Director of Endoscopy Unit, Study performed at the Endoscopy Unit of the Division of Gastroenterology of Universidade Federal de São Paulo, São Paulo, Brazil*

*(Received 1 September 1998)*

**Background: Pancreatic pseudocyst endoscopic drainage has been described as a good treatment option, with morbidity and mortality rates that are lower than surgery. The aim of our study is to describe the efficacy of different forms of endoscopic drainage and estimate pseudocyst recurrence rate after short follow up period.**

**Patients and Methods: We studied 30 patients with pancreatic pseudocyst that presented some indication for treatment: persistent abdominal pain, infection or cholestasis. Clinical evaluation was performed with a pain scale, 0 meaning absence of pain and 4 meaning continuous pain. Pseudocysts were first evaluated by abdominal CT scan, and after endoscopic retrograde pancreatography the patients were treated by transpapillary or transmural (cystduodenostomy or cystgastrostomy) drainage. Pseudocyst resolution was documented by serial CT scans.**

**Results: 25/30 patients could be treated. Drainage was successful in 21 (70% in an 'intention to treat' basis). After a mean follow-up of 42 ± 35.82 weeks, there was only 1 (4.2%) recurrence. A total of 6 complications occurred in 37 procedures (16.2%), and all but 2 were managed clinically and/or endoscopically: there was no mortality related to the procedure. Patients submitted to combined drainage needed more procedures than the other groups. There was no difference in the efficacy when we compared the three different drainage methods.**

**Conclusions: We concluded that pancreatic pseudocyst endoscopic drainage is possible in most patients, with high success rate and low morbidity.**

*Keywords:* Pancreatic pseudocyst, cystenterostomy, endoscopic drainage, plastic stents

### INTRODUCTION

Pancreatic pseudocysts are collections of pancreatic juice, with high enzymatic concentration, located inside or around the gland, formed as a result of pancreatic inflammation and/or ductal injury [1–3]. They are observed as a complication of acute pancreatitis (16% to 50%), or during the course of chronic pancreatitis, in 20% to 40% of the cases [4, 5].

Most of the pseudocysts secondary to acute pancreatitis resolve spontaneously [6,7] while

*Address for correspondence: Rua Pedro de Toledo, 980, cj 66, CEP 04039-002, São Paulo-SP, Brazil, Tel.: (+5511) 573-9838, Fax: (+5511) 575-3834, e-mail: Angelo@gastro.epm.br those associated with chronic disease are associated with a lower spontaneous resolution rate and more complications [2, 8]. Complications include infection, rupture, haemorrhage, biliary compression with or without jaundice, gastrointestinal obstruction, chronic pain, oesophageal varices, leakage with ascites or pleural fluid and pseudoaneurism [8–10].

Indications for drainage include: lesions larger than 4 cm or present longer than six weeks, persistent pain, complications and increased volume documented by imaging test [6, 7, 11].

Complications of surgical treatment occur in 10% to 30%, mortality in 1% to 5% and recurrence in 10% to 20% of the cases [9, 12, 13].

Percutaneous aspiration is a simple method but associated with frequent recurrence [6, 14]. Percutaneous drainage guided by US or CT is reserved for critically ill patients or for those with infected pseudocyst [15].

Endoscopic drainage has been reported during the last decade with success rates higher than 80% and can be transmural (cystgastrostomy or cystduodenostomy) or transpapillary [11, 12, 16, 17]. Both can be combined although transpallary drainage should be the first option because it's probably associated with a lower complication rate [18].

Endoscopic treatment is reported to have lower morbidity than surgery [5]. This study aimed to evaluate effectiveness and complications of pancreatic pseudocysts endoscopic drainage.

### MATERIALS AND METHODS

Between June 1994 and May 1997, 30 patients referred to our Unit for pancreatic pseudocyst drainage were included in a prospective study. Inclusion criteria were: pancreatic pseudocyst confirmed by abdominal CT, more than 18 years of age, presence of at least one indication for drainage: pseudocyst larger than 4 cm present for at least 6 weeks with persistent abdominal pain, progressive increase in size or complication (gastrointestinal or biliary obstruction, infection). Severity of abdominal pain was recorded before the drainage and at the end of the study according to the following score: zero (no pain), 1(sporadic episodes of pain), 2 (weekly episodes), 3 (daily attacks of pain).

Size, number, location and relationship of the pseudocyst with the stomach or duodenum were evaluated with CT scan. During endoscopy we always attempted to identify any indentation on gastric or duodenal wall possibly caused by the pseudocyst. Retrograde endoscopic cholangiography was obtained whenever cholestasis was present.

According to the findings during ERCP, whenever possible, patients were submitted to endoscopic treatment or referred to surgery. Endoscopic drainage was performed in 4 different ways, as follows:

*Group I – transpapillary drainage* (TP) – presence of communication between the main pancreatic duct (MPD) and the pseudocyst, and we were able to cross any stricture and place a pancreatic plastic stent.

*Group II – cystgastrostomy* (CG) – when transpapillary drainage was not possible (absence of communication between the pseudocyst and MPD, or unable to access the MPD) and there was a clear compression on the gastric wall, and the distance between the pseudocyst and the gastric wall was less than 1 cm measured by CT scan.

*Group III – cystduodenostomy* (CD) – same as described for cystgastrostomy but compression was present on the duodenum.

*Group IV – combined drainage* (CB) – performed whenever the pseudocyst did not heal after a single drainage procedure.

Any treatment was repeated whenever necessary. Transpapillary drainage was the first treatment option. The distal end of the stent was located in the MPD, not necessarily inside the pseudocyst. In the presence of a MPD stricture dilation was performed with a passage dilator and the distal end of the stent was positioned proximally to the stricture. Transmural drainage started with pseudocyst puncture trough the gastric or duodenal wall with the tip of a polipectomy snare. After spontaneous drainage of characteristic fluid, the snare was removed and a guide wire was passed into the snare sheath; then a plastic stent was placed over the wire. Straight or pigtail plastic stents (9 or 10 Fr) were used for transmural drainage, and 5 or 7 Fr plastic pancreatic stents were inserted in transpapillary drainage.

Abdominal pain and obstructive symptoms were evaluated clinically and regression of the pseudocyst was confirmed by CT. Once pseudocyst resolution was confirmed the stents were removed and pancreatogram was done to look for possible ductal lesions.

An intention to treat analysis was done to evaluate our results. Statistical analysis were done with Fisher test, Student "*t*" test, X2 and Kruskal–Wallis, whenever appropriate.

### RESULTS

Most of the patients (90%) were male and age ranged from 24 to 64 years ($x = 38,46 \pm 9,87$ years). Pseudocyst aetiology was alcoholic chronic pancreatitis (ACP) in 26/30 patients (86,7%), abdominal trauma in 2/30 (6,7%) and 2/30 after surgery (6,6%). Three patients with ACP had a history of pseudocyst surgical treatment. Persistent abdominal pain was the most frequent drainage indication (93,4%).

Overall 38 pseudocysts was diagnosed: 24 patients with one lesion, 4 patients with 2 and 2 patient with 3. Pseudocyst size ranged from 2 to 20 cm ($x = 9,14 \pm 4,93$ cm), located in the body (52,6%), head (31,6%) or tail (15,8%).

Indentation of the gastric or duodenal wall was identified in 20/30 patients (66,7%), MPD stricture and communication with the pseudocyst in 57,7% and 60% respectively. Fluid was studied in 17 patients and culture was positive in 6 (35%).

After ERCP, in 5/30 patients (16,7%) there were no favourable conditions for endoscopic drainage because there was no access to the lesion (transpapillary or transmural). In the 25 remaining patients (83,3%) endoscopic drainage was performed: 8 cases in Group I (transpapillary), 5 in Group II (cystgastrostomy), 7 in Group III (cystduodenostomy) and 5 in Group IV (combined drainage), total of 37 procedures (14 CG, 7 CD and 16 TP). The average number was of 1.48 procedures per patient and Group IV had more procedures compared with the other groups ($p = 0,002$).

Non-obstructed stents were removed after an average of $59,10 \pm 28,41$ days (range 27 to 120 days). Main pancreatic duct changes (irregularities and strictures) were noted in 2 patients after transpapillary drainage, both had resolution of the pseudocyst without recurrence.

Success (regression of the pseudocyst) was obtained in 21/25 (84%) in a 'per protocol analysis', but a more realistic intention to treat analysis showed that rate to be of 21/30 patients (70%).

Pseudocyst regression was different according to drainage group. Better results occurred in Group IV, although such difference was not statistically significant (Tab. I).

Twenty one 21 patients were followed from 2 to 124 weeks ($x = 42 \pm 35,82$ weeks) and recurrence was diagnosed in only one patient (4,8%), 12 months after CG. He was then submitted to another CG guided by endoscopic ultrasound and he is now asymptomatic. Only 4 patients are taking non-opioid analgesic medication because of abdominal pain, but with clinical score improvement, and CT didn't show pseudocyst recurrence.

TABLE I Success rate in the different drainage groups

| Patient | N | Success rate (%) |
|---|---|---|
| Transpapillary | 8 | 75 |
| Cystgastrostomy | 5 | 80 |
| Cystduodenostomy | 7 | 85 |
| Combined | 5 | 100 |
| Analysis | | |
| for protocol | 25 | 84 |
| intention to treat | 30 | 70 |

TABLE II Frequency of complications (%) in the different groups

| Drainage | Complications | % |
|---|---|---|
| Transpapillary | pancreatitis | 2.7 |
| Cystgastrostomy | stent proximal migration bleeding* | 5.4 |
| Cystduodenostomy | perforation duodenal* | 2.7 |
| Combined | stent proximal migration pneumoperitoneum | 5.4 |
| Total | | 16.2 |

* referred to surgery.

Evaluation of abdominal pain in the end of the follow up period identified 17 patients without pain (score zero) and four patients with ACP still complain of some pain. The average abdominal pain score was $2.48 \pm 0.51$ before and $0.28 \pm 0,64$ after treatment ($p < 0,001$, 95% Cl: 1.83–2.55).

We analysed in an univariate way some factors that could be predictive of a good endoscopic drainage result: gastroduodenal indentation, MPD stricture, communication between the pseudocyst and MPD, age, size of the pseudocyst, cholestasis, presence of multiple lesions, pseudocyst larger than 6 cm, serum albumin < 3,5 g/dl. Only the presence of gastroduodenal wall indentation seen at endoscopic reached statistical significance ($p = 0,03$).

The complications of the 37 procedures occurred after CG (one case of each: bleeding, asymptomatic pneumoperitoneum and proximal stent migration), TP (one case of each: mild pancreatitis and proximal stent migration) and CD (duodenal perforation in one case), total of 6/37 (16.2%) complications (Tab. II). Most complications were benign and managed clinically and/or endoscopically. Two patients (bleeding and perforation) needed surgery (5,4%). There was no mortality related to the procedure. There was no difference between treatment Groups.

### DISCUSSION

There is still a lot of controversy regarding pancreatic pseudocysts treatment, and up to now there is not an ideal way to treat them.

There is no consensus in the literature regarding antibiotic use before pseudocyst endoscopic drainage. Some authors [11, 13] recommended its use while others [17, 18] just indicated it when there are evidences of pseudocyst infection. In this study we only prescribed parenteral antibiotics when there were clinical signs of infection and/or positive cultures, or when we were not able to drain the lesion or the bile duct (when biliary stricture was present). Pseudocyst infection was not among our complications. All 6 patients with a positive pseudocyst fluid culture had resolution without recurrence, and if drainage through the stent is effective, pseudocyst infection doesn't seem to interfere with the result of endoscopic treatment, as observed in this study.

Our findings during pancreatograms were similar with those described earlier authors [4, 17, 18].

Several complications of transpapillary drainage have been described and they include: stent occlusion (symptomatic or not), stent proximal or distal migration, pseudocyst infection, pseudocyst recurrence or worsening, pancreatitis, pancreatic ducts inflammatory changes and duodenal erosions [12, 19–21]. Stent removal or exchange should be done within 4 to 6 weeks due to high occlusion rate after this period [22], up to 100% after 9 weeks [23]. In our study 3 patients needed stent exchange because of occlusion, but none of them presented any symptoms and those were not considered complications. One patient had a transpapillary stent proximal migration and it was not possible to remove it despite pancreatic sphincterotomy and multiple attempts. He is asymptomatic and without pseudocyst recurrence after a 29 month follow up. Some authors had suggested that such complication doesn't have any clinical consequence [12, 22]. Distal migration is more common, doesn't cause adverse effects and should not be considered as a complication [22]. There was one case of mild pancreatitis that responded to clinical measures as reported earlier [17, 22].

Pancreatic stents can induce MPD changes like irregularities and strictures, and also changes in secondary branches, similar those changes found in chronic pancreatitis [19, 24]. They may be asymptomatic and reversible with stent removal or exchange. We noticed morphologic changes on pancreatic ducts after transpapillary drainage in 2 cases, and both were asymptomatic and had no recurrence. We should keep in mind remind that such changes can also be part of the natural history of chronic pancreatitis.

Transmural drainage, mainly CG, presents higher risk of complications, specially bleeding [13, 14, 18]. We had 3 complications after CG (bleeding, pneumoperitoneum and stent migration into the pseudocyst) and one after CD (perforation). Asymptomatic pneumoperitoneum was diagnosed one week after the procedure during a routine CT. Possibly, it was due to a small fistula during CG, maybe secondary to the presence of ascites, even though previous CT scan showed that the distance between gastric wall and the pseudocyst was less than 1 cm [25]. There was no difference regarding the frequency of complications in the different Groups (Tab. II), although both complications that needed surgery (bleeding and duodenal perforation) occurred after transmural drainage.

Endoscopic ultrasound (EUS) allows identification of the best puncture site, specially in cases without a clear gastroduodenal indentation, besides identifying vascular structures between gastric or duodenal wall and the pseudocyst increasing effectiveness and safety of endoscopic drainage [26, 27]. During the study EUS was not available at our institution, and maybe both complications that needed surgery (bleeding and perforation) could be avoided, besides allowing drainage in those cases in which we were unable to identify gastroduodenal compression. More recently we started to perform EUS guided transmural drainage whenever gastric or duodenal wall is not detected during endoscopy [28]. Our success (pseudocyst regression) rate was similar to other published series [12, 14, 17]. There was no difference among different Groups, suggesting that drainage route had no influence on the results.

Our recurrence rate was also comparable to those reported by other authors [11, 14]. The only patient that presented pseudocyst recurrence was treated with another endoscopic drainage, as already described [14, 18].

Comparative evaluation of abdominal pain score before and after drainage showed statistically significant improvement. Asymptomatic patients were mainly those in alcohol abstinence, and probably this an important factor for good clinical response during long follow up.

In conclusion, we believe that pancreatic pseudocyst endoscopic drainage is possible in most patients, with high success rate and low morbidity. Most complications can be clinically and/or endoscopically managed. Transpapillary drainage should be tried before transmural drainage, because it is associated with low morbidity. Patients that are successfully drained have important clinical improvement and recurrence is uncommon, specially for those that stay in alcohol abstinence.

***Acknowledgments***

This study was financially supported by Fundação de Amparo à Pesquisa do Estado de São Paulo (FAPESP), process # 93/3398-0.

***References***

[1] Bourliere, M. and Sarles, H. (1989). Pancreatic cysts and pseudocysts associated with acute and chronic pancreatitis. *Dig. Dis. Sci.*, **34**, 343–348.
[2] Sahel, J. (1991). Endoscopic drainage of pancreatic cysts. *Endoscopy*, **23**, 181–184.
[3] Banks, P. A. (1997). Practice guidelines in acute pancreatitis. *Am. J. Gastroenterol*, **92**, 377–386.
[4] Barthet, M., Bugallo, M., Moreira, L. S., Bastid, C., Sastre, B. and Sahel, J. (1993). Management of cysts and pseudocysts complicating chronic pancreatitis. The retrospective study of 143 patients. *Gastroenterol Clin. Biol.*, **17**, 270–276.

[5] Lawson, J. M. and Baillie, J. (1995). Endoscopic therapy goes pancreatic pseudocysts. *Gastrointest Endosc Clin. N. Am.*, **5**, 181–193.

[6] Grace, P. A. and Williamson, R. C. N. (1993). Modern management of pancreatic pseudocysts. *Br. J. Surg.*, **80**, 573–581.

[7] Huibregtse, K. and Smits, M. E. (1994). Endoscopic management of diseases of the pancreas. *Am. J. Gastroenterol*, **89**, S66–S77.

[8] Bradley III, E. L. (1989). Complications of chronic pancreatitis. *Surg. Clin. N. Am.*, **69**, 481–497.

[9] Adams, D. B. and Anderson, M. C. (1992). Changing concepts in the surgical management of pancreatic pseudocysts. *Am. Surg.*, **58**, 173–180.

[10] Yeo, C. J., Bastidas, J. A., Lynch-Nyhan, A., Fishman, E. K., Zinner, M. J. and Cameron, J. L. (1990). The natural history of pancreatic pseudocysts documented by computed tomography. *Surg. Gynecol Obstet*, **170**, 411–417.

[11] Catalano, M. F., Geenen, J. E., Schmaiz, M. J., Johnson, G. K., Dean, R. S. and Hogan, W. J. (1995). Treatment of pancreatic pseudocysts with ductal communication by transpapillary pancreatic duct endoprosthesis. *Gastrointest Endosc*, **42**, 214–218.

[12] Smits, M. E., Rauws, E. A. J., Tytgat, G. N. J. and Huibregtse, K. (1995). The efficacy of endoscopic treatment of pancreatic pseudocysts. *Gastrointest Endosc*, **42**, 202–207.

[13] Kozarek, R. A. (1997). Endoscopic treatment of pancreatic pseudocysts. *Gastrointest Endosc Clin. N. Am.*, **7**, 271–283.

[14] Cremer, M., Deviere, J. and Engelholm, L. (1989). Endoscopic management of cysts and pseudocysts in chronic pancreatitis: long-term follow-up after 7 years of experience. *Gastrointest Endosc*, **35**, 1–9.

[15] Servant, E., DeStefano, A., Weiner, T. M. and Jaques, P. F. (1992). Long term results of percutaneous catheter drainage of pancreatic pseudocysts. *Surg. Gynecol Obstet*, **175**, 293–298.

[16] Kozarek, R. A., Ball, T. J., Patterson, D. J., Freeny, P. C., Ryan, J. A. and Traverso, W. (1991). Endoscopic transpapillary therapy goes disrupted pancreatic duct and peripancreatic fluid collections. *Gastroenterology*, **100**, 1362–1370.

[17] Barthet, M., Sahel, J., Bertei, C. B. and Bernard, J. P. (1995). Endoscopic transpapillary drainage of pancreatic pseudocysts. *Gastrointest Endosc*, **42**, 208–213.

[18] Binmoeller, K. F., Seifert, H., Walter, A. and Soehendra, N. (1995). Transpapillary and transmural drainage of pancreatic pseudocytes. *Gastrointest Endosc*, **42**, 219–224.

[19] Sherman, S., Alvarez, C., Robert, M., Ashley, S. W., Reber, H. A. and Lehman, G. A. (1993). Polyethylene pancreatic duct stent-induced changes in the normal dog pancreas. *Gastrointest Endosc*, **39**, 658–664.

[20] Ponchon, T., Bory, R. M., Hedelius, F., Roubein, L. D., Paliard, P., Napoleon, B. and Chavaillon, A. (1995). Endoscopic stenting goes pain relief in chronic pancreatitis: results of the standardized protocol. *Gastrointest Endosc*, **42**, 452–456.

[21] Smits, M. E., Badiga, S. M., Rauws, E. A. J., Tytgat, G. N. J. and Huibregtse, K. (1995). Long-term results of pancreatic stents in chronic pancreatitis. *Gastrointest Endosc*, **42**, 461–467.

[22] Siegel, J. and Veerappan, A. (1991). Endoscopic management of pancreatic disorders: potential risks of pancreatic prostheses. *Endoscopy*, **23**, 177–180.

[23] Ikenberry, S. O., Sherman, S. and Hawes, R. H. (1994). The occlusion rate of pancreatic stents. *Gastrointest Endosc*, **40**, 611–613.

[24] Kozarek, R. A. (1990). Pancreatic stents can induce ductal changes consistent with chronic pancreatitis. *Gastrointest Endosc*, **36**, 93–95.

[25] Della Libera, E. and Ferrari, A. P. (1997). Asymptomatic pneumoperitoneum after endoscopic treatment of pseudocysts. *Endoscopy*, **29**, 231–232.

[26] Binmoeller, K. and Soehendra, N. (1995). Endoscopic ultrasonography in the diagnosis and treatment of pancreatic pseudocysts. *Gastrointest Endosc Clin. N. Am.*, **5**, 805–816.

[27] Chan, A. T., Heller, S. J., Vam Dam, J., Carr-Locke, D. L. and Banks, P. A. (1996). Endoscopic cystgastrostomy: roll of endoscopic ultrasonography. *Am. J. Gastroenterol*, **91**, 1622–1625.

[28] Ardengh, J. C., Della Libera, E. and Ferrari, A. P. (1998). Endosonography-guided drainage of pancreatic pseudocyst without gastric or duodenal compression. *Endoscopy*, **30**, S71–2.

